# Local α-receptor antagonists as a targeted therapeutic approach in patients with vasopressor-induced symmetrical peripheral gangrene

**DOI:** 10.1515/iss-2025-0044

**Published:** 2026-01-07

**Authors:** Philipp Edmund Lamby, Vinzent Forstmeier, Lukas Prantl, Michael Pawlik, Jasmin Lenhard

**Affiliations:** Klinik für Plastische, Ästhetische, Hand- und Wiederherstellungschirurgie, Klinikum Passau, Innstraße 76, 94032 Passau, Germany; Klinik für Unfallchirurgie und Orthopädie, Rekonstruktive und Septische Chirurgie, Bundeswehrkrankenhaus Ulm, Oberer Eselsberg 401, 89081 Ulm, Germany; Abteilung für Plastische, Hand- und Wiederherstellungschirurgie, Universitätsklinikum Regensburg, Franz-Josef-Strauß-Allee 11, 93053 Regensburg, Germany; Klinik für Plastische, Hand- und Wiederherstellungschirurgie, Caritas Krankenhaus St. Josef, Landshuter Straße 65, 93053 Regensburg, Germany; Klinik für Anästhesiologie, Intensiv- und Notfallmedizin, Caritas Krankenhaus St. Josef, Landshuter Straße 65, 93053 Regensburg, Germany

**Keywords:** symmetrical peripheral gangrene, phentolamine, alpha-adrenergic blockade, vasopressor complications, digital ischemia

## Abstract

**Objectives:**

Symmetrical peripheral gangrene (SPG) is a rare condition characterized by symmetrical gangrene affecting the acral regions of two or more extremities, without the presence of major vessel obstruction. The precise pathophysiology of SPG is unclear, but it is believed to involve various biochemical pathways and is often associated with vasopressor therapy. Despite various therapeutic attempts, there is no established standard treatment for SPG.

**Methods:**

This study evaluated the efficacy of locally administered phentolamine, an alpha-receptor antagonist, in four critically ill patients undergoing catecholamine therapy and presenting with SPG. Tissue perfusion was assessed using clinical observation and indocyanine green-assisted laser angiography before and after phentolamine administration.

**Results:**

All four patients demonstrated immediate improvement in tissue perfusion following phentolamine application, as evidenced by angiography. No amputations were necessary. The patients’ clinical outcomes suggest that phentolamine effectively counteracts vasopressor-induced ischemia.

**Conclusions:**

Phentolamine offers a targeted therapeutic approach for vasopressor-induced SPG by mitigating vasoconstriction without systemic side effects. Its incorporation into clinical practice requires further validation to optimize its application and efficacy.

## Introduction

Symmetrical peripheal gangrene (SPG) was first described by J. Hutchinson in 1891 [1], 2]. It is characterized as symmetrical gangrene of acral areas in two or more extremities without vasculitis or major vessel occlusion [3], 4]. SPG may lead to tissue necrosis and as a consequence often digit or limb amputation becomes necessarily. Furthermore, this clinical syndrome leads to a significant increase of morbidity and mortality [5]. Overall it is a rare medical condition and only a few case series have been reported [6].

The detailed pathophysiology of SPG is still unclear and it is supposed that it is affected by various biochemical pathways. It may occur as a result of the Shwartzman phenomenon. This means that endotoxins are released in the context of bacterial infection. These endotoxins contain lipopolysaccharides leading to platelet plugging in peripheral arterioles [2]. Another pathway supposed to promote SPG is disseminated intravascular coagulation (DIC). DIC means a systemic activation of the coagulation system leading to microvascular thrombosis and major haemorrhage at the same time. Inappropriate activation of thrombin induces conversion of fibrinogen to fibrin, activation and consumption of platelets, activation of endothelial cells and fibrinolysis [7], 8]. The clinical presentation of DIC is variable depending on the balance between clot formation and severity of consumption of coagulation factors and platelets [9]. Furthermore, it is assumed that vasoactive drugs are highly involved in the development of SPG as the potential vasoconstrictive side effect of these drugs is documented in many reports [10]. Vasopressors play an important role in managing hemodynamically unstable hypotensive patients, including critically ill patients with cardiogenic or septic shock. Most vasopressors impact the contraction of peripheral blood vessels, potentially leading to peripheral ischemic conditions and contributing significantly to the development of systemic peripheral gangrene [11]. In addition to that, SPG may also occur as a result of different infectious diseases (e. g. measles, chickenpox), malignancy, ergotism or disorders of the coagulation cascade (e. g. protein C deficiency, protein S deficiency, antithrombin III deficiency) [12].

Although various therapeutic approaches (e. g. hyperbaric oxygen therapy, anti-platelet medication, application of sildenafil citrate) have been described in the literature, there is no established standard treatment so far [7], 12], 13]. In 2003 Tefor Nodwell and Don Lalonde were able to show that adrenaline-induced vasoconstriction can be reversed by application of phentolamine. Phentolamine is a competitive alpha-receptor antagonist and normally used as antihypertensive drug [14]. As vasoactive drugs may play an important role in the development of SPG the aim of this paper is to investigate the use of phentolamine as an antidot for SPG.

## Patients and methods

This trial was conducted in the context of an alternative healing attempt. It was approved by the Ethics Committee of the University of Regensburg (reference number: 25-4042-104) and was planned in accordance with the Declaration of Helsinki 2013. The aim of the study was to prevent necrosis or amputation of digits due to SPG by the local application of an alpha-receptor antagonist called Phentolamine.

Included in the study were critically ill patients who received adrenaline to maintain circulation and who showed reduced perfusion of acral areas in the sense of SPG. Patients without catecholaminergic circulatory support as the cause of ischemic digits and known hypersensitivity or allergy to phentolamine were excluded. Also, patients with local infections in the region of the ischemic acral areas were excluded, as local inflammation could alter microcirculatory perfusion and thus confound the interpretation of phentolamine efficacy.

As mentioned above, phentolamine was utilized to prevent further progress of the SPG and applicated locally to avoid amputation of digits. Phentolamine is a competitive alpha-receptor antagonist and was first introduced in 1952. It has initially been used as an antihypertensive agent as well as for the clinical management of accidental extravasation of endogenous catecholamines. In the field of dentistry, the FDA approved its use in 2008 for the reversal of local anesthesia, marketed under the name “OraVerse” [14], 15]. According to manufacturer guidelines, an intravenous dose of 3–5 mg is recommended to reduce blood pressure. For the reversal of epinephrine-induced subcutaneous vasospasm, the recommended dosage is 5–10 mg of phentolamine diluted in 10 mL of saline, administered subcutaneously at the site of epinephrine infiltration [14], 16]. For our study, we selected a higher concentration of 1 mg/mL phentolamine.

In this study, tissue perfusion was assessed both clinically and through fluorescence angiography. This non-invasive technique uses the fluorescent dye indocyanine green (ICG), which is administered intravenously. ICG distributes through the bloodstream, primarily binding to plasma proteins. Upon exposure to near-infrared laser light, the dye is excited to emit fluorescence, which is then used to visualize tissue perfusion in real time. Fluorescence occurs when the dye’s electrons are elevated to a higher energy state by laser excitation and then return to their ground state, releasing energy as fluorescence. The absorption spectrum of ICG ranges from 750 to 800 nm, with a peak emission wavelength around 830 nm in the near-infrared range [17], [18], [19]. In this study, fluorescence angiography was performed using the SPY-Elite^®^ Fluorescence Imaging System, which allows for visualization of the microvascular blood flow to a depth of 7–10 mm. This system, consisting of a CCD camera, a data processing unit, and two monitors, provides mobile and flexible positioning during examination. The camera is mounted on an articulated arm and incorporates both a laser light source and a distance sensor that ensures optimal proximity to the patient’s skin. Following the administration of ICG, the SPY-Elite^®^ system allows real-time visualization of tissue perfusion, with better-perfused areas appearing brighter and poorly perfused regions darker. The recordings were captured over a period of 136 s with a frame rate of 7.5 frames per second [20], 21]. For fluorescence angiography in this study, the dye Verdye^®^, which contains ICG, was used. ICG, a fluorescent water-soluble tricarbocyanine with a molecular weight of 774.96 Da, binds to plasma proteins, such as albumin, with 98 % affinity after intravenous administration. Only 2 % remains unbound in serum and is rapidly cleared through the liver and biliary system. Due to its physical and chemical properties, including deep tissue penetration of near-infrared light and high contrast imaging, ICG is well-suited for fluorescence angiography [19], 22].

The diagnosis SPG was made on the basis of clinical criteria such as livid skin discoloration and incipient skin necrosis on both upper/lower extremities or other acral areas. After the diagnosis was made, an ICG-supported laser angiography was performed to evaluate perfusion. In all patients, major vessel patency was confirmed by palpation of peripheral pulses and, when indicated, Doppler ultrasonography. ICG angiography demonstrated intact macrovascular perfusion but severe acral hypoperfusion, consistent with microcirculatory ischemia typical of SPG. Subsequently, 1 mg phentolamine was applied locally to the critically affected area of the ischemic acres. Therefore, we used phentolamine (Regitin^®^) from the manufacturer Novartis (Basel, Switzerland), supplied as 10 mg/mL ampoules. The solution was diluted with 0.9 % sodium chloride to achieve a final concentration of 1 mg/mL. A dose of 1 mg phentolamine was administered subcutaneously per injection site using a 27-gauge needle. This was repeated as necessary until the ischemia regressed. The highest cumulative dose administered to a single patient was 7 mg, distributed across three extremities. For anatomical orientation during local injection, the standardized zone allocation for digital application described by Lalonde and Nodwell was followed wherever possible. According to this scheme, injections are placed at three key sites along the digit: the volar distal palmar crease, the volar base of the proximal phalanx, and the volar base of the middle phalanx [14]. Laser-assisted fluorescein angiography was then performed again 10 min after application of phentolamine to assess perfusion.

**Figure 1:**
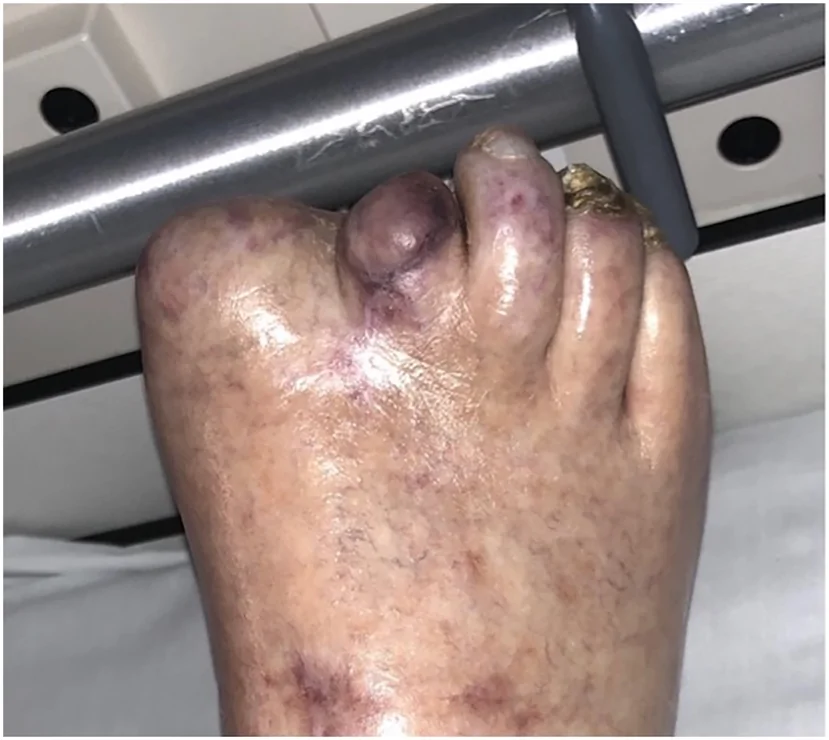
Clinical finding before application of phentolamine.

**Figure 2:**
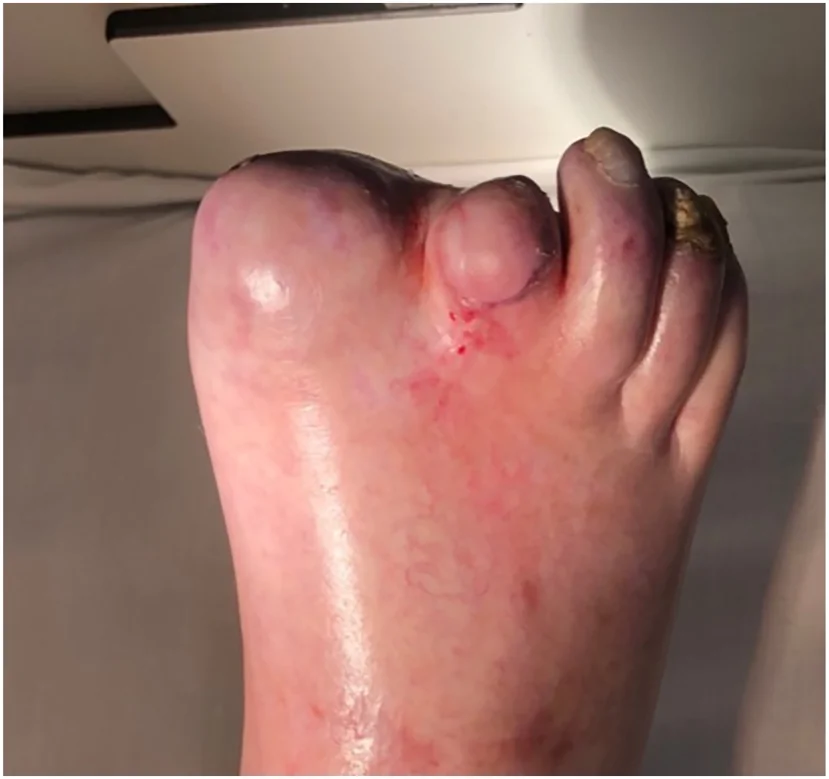
Clinical finding after application of phentolamine.

**Figure 3:**
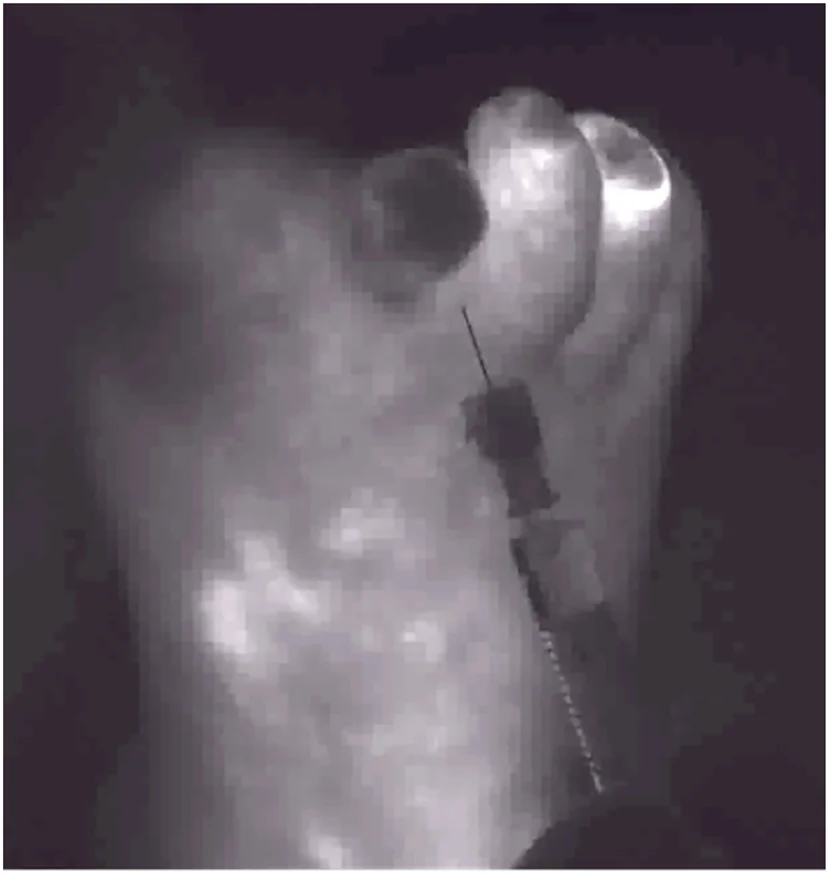
ICG angiography before application of phentolamine.

**Figure 4:**
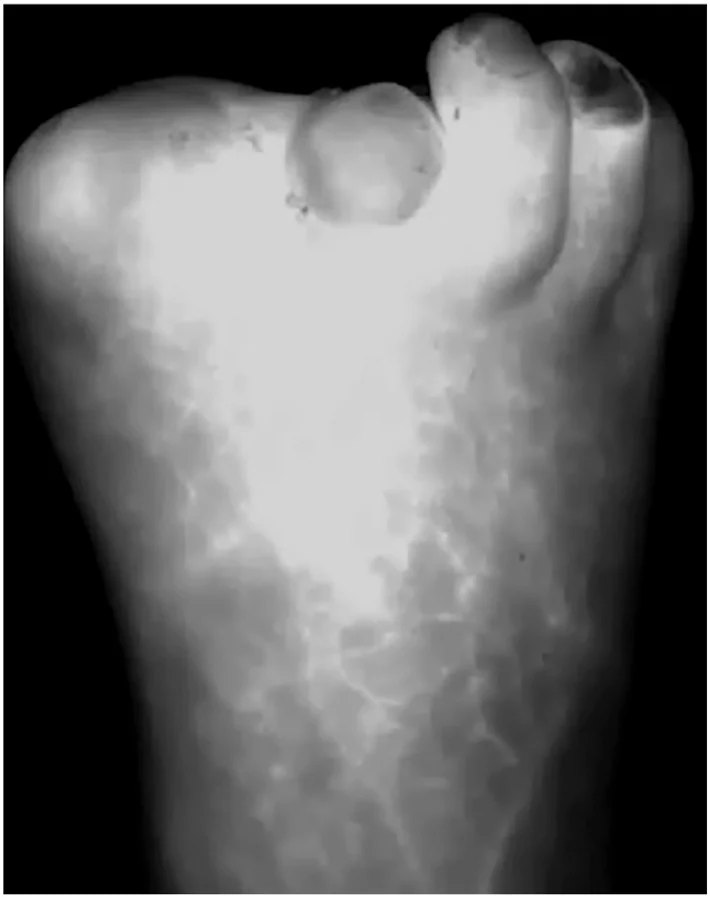
ICG angiography after application of phentolamine.

An example of the clinical findings, along with an ICG angiography before and 10 min after the application of phentolamine, is shown below (Figures 1–4).

## Results

In total four patients were included. The observation period ran from February 2018 to October 2018. All patients were treated at the intensive care unit (ICU) of the Caritas Hospital St. Josef Regensburg. The mean patient age was 74.25±9.53 years, the mean BMI was 22.99±8.84 kg/m^2^. The relevant epidemiological and patient-related data are listed in Table 1 below.

**Table 1:** Overview of patient data.

Patient number	Sex	Age, years	BMI, kg/m^2^	Reason for admission/ICU treatment
01	Male	63	12.14	Sepsis (due to pneumonia)
02	Female	70	31.25	Urosepsis (due to perinephritic abscess on the left side)
03	Female	84	29.07	Metabolic disorder due to acute renal failure
04	Female	80	19.53	Cholangiosepsis (most likely due to pancreatic carcinoma)

All four patients showed livid discoloration or incipient necrosis of acral areas in the sense of SPG. Local application of phentolamine was therefore carried out in all four patients. All patients showed an improvement in perfusion immediately after administration of phentolamine, both clinically and in the laser angiography. In two patients, reperfusion persisted without recurrence, while in the remaining two, the phentolamine application was repeated due to partial relapse of ischemia. None of the patients required amputation of digits. None of the patients experienced a drop in systemic blood pressure while taking phentolamine, nor was an increase in the catecholamine dose necessary. On average, the patients were treated in the ICU for 19±13.46 days. However, it must be mentioned that one patient died three days after admission in the ICU. The other three patients were transferred to normal surgery or internal medicine ward once their general condition had improved. The dose used of phentolamine and the localization are listed in the following Table 2.

**Table 2:** Overview of results.

Patient number	Most affected area	Peak dose of catecholamines, mg/h	Dose of phentolamine administered, mg	Amputation	Duration of ICU treatment, days
01	Right foot (D1, D2, D3)	Noradrenaline: 4.3 Dobutamine: 10.0	1	No (but patient died three days after admission)	3
02	Right foot (D2, D3)	Noradrenaline: 1.9 Dobutamine: 10.0	2	No	35
03	Left hand (D2, D3, D4, D5), left foot (D1, D2), right foot (D1, D2, D3, D4), right hand (back of the hand)	Noradrenaline: 2.4 Dobutamine: 20.0	7	No	15
04	Right hand (D2)	Noradrenaline: 7.6 Dobutamine: 10.0	1	No	23

To illustrate the coagulation status and disease severity at the time of SPG diagnosis, key laboratory parameters were collected for each patient. Platelet count, lactate levels, and SOFA score at the time of SPG diagnosis are summarized in Table 3 below. If the exact time of diagnosis was not documented, the closest available values to the administration of phentolamine were used. D-dimer levels were unfortunately not available for these patients.

**Table 3:** Key laboratory results.

Patient number	Platelet count, per nanoliter	Lactate level, mmol/l	SOFA-score, points
01	424	8.7	11
02	75	0.9	10
03	58	1	8
04	72	0.8	7

Statistical analysis was limited to descriptive reporting, as no comparative or inferential statistical testing was applicable due to the small sample size.

## Discussion

SPG is a rare medical syndrome that causes symmetrical gangrene of the distal extremities and acral regions [3], 4]. It may appear as a consequence of the Shwartzman phenomenon, DIC or administration of vasoactive drugs [2], 10]. Vasopressors influence the constriction of peripheral blood vessels, potentially triggering peripheral ischemic conditions and may lead to the onset of SPG [11]. The aim of this clinical trial was to investigate if SPG and necrosis of the acral regions can be averted in patients with catecholamine therapy by drug-based local alpha-receptor blockade. For this investigation, individuals afflicted with systemic peripheral gangrene underwent local application of phentolamine, with a dosage of 1 mg per injection site. Subsequently, perfusion of the affected extremity was assessed employing ICG-assisted laser angiography [11].

When using phentolamine as an antidote for vasopressors, the specific mechanisms of action of the various vasopressors must be taken into account. In particular, the receptors through which the various drugs mediate their effect must be considered. Noradrenaline primarily targets alpha receptors, with minimal activity on beta receptors [23]. Dopamine demonstrates dose-dependent effects, causing vasoconstriction at higher doses through activation of alpha receptors [24]. Epinephrine acts on both alpha- and beta-adrenergic receptors. At low doses, it predominantly activates beta receptors, whereas higher doses result in selective activation of alpha receptors [25]. Dobutamine acts on alpha-1, beta-1, and beta-2 receptors, with its effects on alpha-1 receptors (vasoconstriction) balanced by its effects on beta-2 receptors (vasodilation) in blood vessels [26]. Vasopressin on the other hand exerts antidiuretic effects via V2 receptors in the renal collecting ducts and induces vasoconstriction at higher concentrations through binding to V1 receptors. Phentolamine, by blocking alpha-adrenergic receptors, could mitigate the vasoconstrictive effects of norepinephrine, dopamine, and epinephrine in affected tissues. In this study all patients received pharmacological treatment with noradrenaline and dobutamine to achieve hemodynamic stabilization. Clinical manifestations of SPG were observed in all four cases. Following local phentolamine injection, none of the patients required amputation. This targeted blockade is particularly advantageous, as systemic alternatives are often limited in critically ill patients reliant on vasopressors to maintain hemodynamic stability.

There is a lack of consensus regarding the optimal maximum dosage and duration of vasopressor therapy for the treatment of hemodynamically unstable patients. The guidelines of the Survival Sepsis Campaign solely advocate for dopamine and norepinephrine as the primary vasopressors for addressing hypotension in septic shock for maintaining a mean arterial pressure (MAP) of at least 65 mm Hg [27].

As already mentioned above the effect of dopamine depends on the applicated dose. Low doses (1–2 μg/kg/min) cause vasodilatation, medium doses (2–10 μg/kg/min) result in increased cardiac output (via β1-receptors) and high doses (above 10 μg/kg/min) lead to vasoconstriction (via α-receptors) [10]. Kwon et al. demonstrated that the incidence of SPG is more influenced by the peak dose rather than the duration of treatment. Upon adjusting for patient weight, the peak dose of noradrenaline, dopamine, or epinephrine emerged as a significant factor impacting the occurrence of SPG. Conversely, the peak dose of dobutamine or vasopressin showed no significant effect on SPG [11]. In the present study, the lowest peak dose of noradrenaline used was 1.9 mg/h (patient weight: 80 kg) and the highest peak dose of noradrenaline used was 7.6 mg/h (patient weight: 50 kg). Since clinical signs of SPG were observed in all patients, this indicates that SPG can already occur at these doses. Findings by Kwon et al. suggest that limiting the peak dose of norepinephrine and epinephrine may serve as a crucial preventive strategy. Close monitoring of these doses could help reduce the risk of SPG. In patients requiring high doses of vasopressors, prophylactic or therapeutic use of phentolamine may complement stringent dose management. The dose-dependent effects documented by Kwon et al. support the hypothesis that vasopressor-induced vasoconstriction represents a threshold phenomenon. Once a critical dose is exceeded, uncontrolled vasoconstriction ensues, leading to irreversible perfusion deficits and tissue necrosis. These findings underscore the potential role of phentolamine, not only as a therapeutic agent but also as a preventive tool in high-risk scenarios. Moreover, the results indicate that not all vasopressors carry the same risk of SPG. Dobutamine and vasopressin, which do not significantly contribute to SPG development, may be considered as alternatives, particularly in situations where the risk of SPG is elevated.

Beyond dose optimization, future studies should also address the pharmacokinetics and tissue distribution of locally applied phentolamine in ischemic tissue. In this trial, only immediate post-injection perfusion changes have been documented, which limits our understanding of the duration and consistency of the drug’s vasodilatory effect. It remains unclear how long phentolamine remains active in compromised acral tissues, especially given the altered vascular permeability and reduced perfusion in necrotic or pre-necrotic areas. Data from dental trials involving phentolamine mesylate provide valuable reference points: they show that phentolamine significantly reduces the duration of soft tissue anesthesia. These findings suggest a rapid onset and relatively short duration of action in well-perfused tissue [15], 28]. In our cohort, the clinical course further suggests that the duration of phentolamine’s vasodilatory effect may extend beyond what is expected from its known systemic half-life. While the existing literature indicates a rapid onset but relatively short duration, our findings showed that two patients maintained stable reperfusion without recurrence, whereas two others demonstrated only a partial relapse that required a second injection. This pattern implies that, in ischemic acral tissues, local pharmacodynamics may differ substantially from those observed in non-ischemic regions. Specifically, reduced clearance and altered diffusion gradients in low-flow compartments might prolong receptor occupancy and thereby extend the functional duration of action. The variability observed between patients further highlights that the effective duration in compromised acral tissue is likely influenced by factors such as baseline perfusion, the degree of microvascular obstruction, and tissue viability at the time of intervention. A more granular understanding of these dynamics will be critical for defining standardized re-dosing intervals and for determining which patients benefit most from repeated applications. Serial ICG-angiography, an established method in reconstructive surgery for assessing tissue perfusion, could provide insight into the temporal profile of phentolamine’s effects [29]. A better understanding of the pharmacokinetics, including tissue distribution and duration of action, would enable more precise tailoring of dose intervals and repeated dosing protocols. Without such data, the therapeutic potential of phentolamine may remain underutilized, particularly in early or borderline cases of SPG where timely and sustained microvascular reperfusion could prevent irreversible tissue damage.

The local application of phentolamine offers several potential benefits. By providing targeted action, phentolamine induces local vasodilation without affecting systemic blood pressure. This is particularly advantageous in critically ill patients. Pathophysiologically, blocking α-adrenoceptors is a rational approach as it directly addresses vasoconstriction, a central mechanism in SPG. Intervention during the initial stages of the disease could restore microcirculation and potentially slow the progression to irreversible necrosis. In addition to this approach, recent literature discusses alternative or adjunctive strategies that may support tissue salvage in SPG.

A case series of patients with severe COVID-19 and SPG explored the use of therapeutic anticoagulation with heparin or low-molecular-weight heparin, given the presumed imbalance of procoagulant and anticoagulant pathways and the frequent association with DIC. While heparin is known for its anticoagulant and anti-inflammatory effects, all four patients in that series ultimately died from multi-organ failure, and no clear benefit regarding SPG progression could be demonstrated, highlighting the limitations of anticoagulation alone in this context [30].

A case report published by Alfraij et al. described the use of topical nitroglycerin in an 18-month-old child with SPG secondary to septic shock. In this report, the local application of nitroglycerin ointment resulted in clear clinical improvement in peripheral perfusion and partial reversal of skin discoloration. The therapeutic effect is mediated by local PDE5 inhibition and increased cGMP levels, promoting smooth muscle relaxation and enhanced microvascular flow. Although promising, topical nitroglycerin must be used cautiously due to the risk of systemic hypotension and methemoglobinemia, especially in pediatric patients [31].

Furthermore, a recent review also addressing SPG in the setting of vasopressor therapy discussed hyperbaric oxygen therapy (HBOT) and hemoadsorption (HA) as potential supportive measures. HBOT aims to increase oxygen delivery to ischemic tissues and has been reported to improve acral perfusion and reduce tissue loss in selected cases. HA, as an extracorporeal blood purification technique, may reduce circulating cytokine levels in septic shock, potentially mitigating endothelial injury and halting the progression of gangrene. Despite these emerging therapeutic options, amputation remains the final recourse when tissue necrosis becomes irreversible [32].

In summary, there is currently a lack of evidence-based guidelines for the management of SPG. The generally accepted treatment goals focus on halting disease progression, addressing underlying triggers, preventing secondary infections, and debriding necrotic tissue. The literature presented above underscores the urgent need for a multidisciplinary approach and further research to clarify the optimal role of vasodilator therapies, dose-limiting strategies, and targeted alpha-receptor blockade in the prevention and management of vasopressor-induced SPG.

However, this study has several important limitations. Its design as a single-centre, uncontrolled case series with a limited number of patients inevitably introduces a high risk of selection and spectrum bias, meaning that the findings must be viewed as exploratory and hypothesis-generating. All included patients received both noradrenaline and dobutamine, which limits the applicability of these results to other patient groups receiving different vasopressor regimens. Additionally, spontaneous improvement of peripheral perfusion cannot be entirely excluded, nor can the possible effects of supportive measures such as physical measures (e. g. topical warming), the discontinuation of vasopressors, or parallel anticoagulation therapy. A further limitation is the exclusion of an early non-survivor, which may have introduced survivorship bias. Another methodological consideration concerns the question of why no intraindividual comparison between extremities was performed. Although SPG is often described as a bilaterally distributed condition, clinical experience and published case series consistently show that the extent and rate of ischemic progression differ markedly between limbs [33]. As a result, using one extremity as an internal control would not have produced valid comparative data, as the pathological course is rarely symmetrical in severity or timing. Furthermore, the rapid clinical deterioration characteristic of SPG often requires immediate intervention, leaving insufficient time to establish a standardized or ethically acceptable intraindividual delay before treatment on the contralateral side. These factors made a bilateral controlled design unfeasible despite the generally symmetrical distribution pattern of SPG. Moreover, while the local injection of phentolamine appears promising for early intervention, its benefit is likely negligible once necrosis has progressed beyond a reversible stage. In practical terms, the need for careful injection technique, precise dosing, and the absence of standardized protocols currently restrict broader use. Finally, limited data on the local pharmacokinetics and distribution in ischemic tissues hinder the development of clear treatment schedules for repeat applications. To enhance transparency and help assess external validity, we have added key laboratory parameters to the Methods section. These include platelet count, lactate levels, and SOFA scores at the time of SPG diagnosis. Although D-dimer levels were not available for these cases, the additional data provide a clearer picture of each patient’s coagulation status and disease severity. Despite these constraints, the results with regredience of the SPG in all four patients and lack of need for amputation in all patients suggest that local alpha-receptor blockade with phentolamine may be a feasible supportive measure to prevent irreversible tissue loss in the early stages of vasopressor-induced SPG. Clinically, its targeted effect and minimal systemic impact could make it a valuable addition to individualized treatment plans, provided that rigorous vasopressor management and close monitoring are ensured.

Before outcome-powered trials can be considered, future research should first focus on systematic early-phase data collection, including pharmacodynamic assessments and objective perfusion monitoring such as serial ICG angiography. These foundational data will be crucial for defining meaningful clinical endpoints, identifying subgroups most likely to benefit, and establishing parameters that could eventually guide standardized treatment protocols.

## Conclusions

SPG represents a rare but serious clinical condition, often precipitated by vasopressors used in critically ill patients. This study examines the potential utility of phentolamine as a locally applied antidote to mitigate vasopressor-induced vasoconstriction and prevent irreversible tissue damage. The observed improvement in perfusion following phentolamine administration, coupled with the absence of amputations in all patients, highlights its potential as a targeted therapeutic intervention. While these findings are promising, larger-scale randomized controlled trials are essential to establish its efficacy, refine dosing protocols, and confirm its role in clinical practice. Further investigations may also explore preventive strategies, including alternative vasopressor regimens, to minimize the risk of SPG. Moreover, additional research is essential to deepen our understanding of the pathophysiology of SPG and to develop evidence-based treatment strategies tailored to its underlying mechanisms [13].
